# The pros of drinking: how the drive for social group membership precipitates alcohol use among young adults

**DOI:** 10.1186/1940-0640-7-S1-A44

**Published:** 2012-10-09

**Authors:** Cydney Dupree, Molly Magill, Timothy Apodaca

**Affiliations:** 1 Center for Alcohol and Addiction Studies, Brown University, Providence, RI, USA

## 

Scientific research has supported the effectiveness of brief motivational intervention (BMI) to reduce young-adult alcohol use and related consequences. Often, BMI includes a decisional balance component examining the “pros and cons” of alcohol use, which attempts to explore client ambivalence about drinking though a discussion of its good (and not-so-good) aspects. The aim of this qualitative study was to investigate the link between the need for social inclusion and engagement in alcohol-related behavior. Participants included 28 young adult (18-24 years old) alcohol users enrolled in a large hospital-based clinical trial. All BMIs were recorded and transcribed, and results were analyzed using NVIVO 9.0 software. Responses to the pros-and-cons dialogue were coded and thematically organized using grounded theory methods. The results identify the role of social group membership in alcohol use, including the need to avoid social exclusion and the desire to facilitate social interaction. Alcohol consumption allowed for such facilitation by providing an “escape” from social anxiety and promoting social behavior. Patterns of alcohol use as influenced by social factors may depend on rejection sensitivity, suggesting that an emphasis on social skills and abstinence-supportive relationships can be used to assist young people trying to reduce alcohol consumption. Social-skills training may prove useful as a preventative measure as well. If young adults feel more confident socially, they may feel less of an impulse to drink to increase the likelihood of social group acceptance.

